# Advancing Dimethacrylate Dental Composites by Synergy of Pre-Polymerized TEGDMA Co-Filler: A Physio-Mechanical Evaluation

**DOI:** 10.3390/biomimetics8080577

**Published:** 2023-12-01

**Authors:** Ali Alrahlah, Rawaiz Khan, Abdel-Basit Al-Odayni, Waseem Sharaf Saeed, Leonel S. Bautista, Ibraheem A. Alnofaiy, Merry Angelyn Tan De Vera

**Affiliations:** 1 Restorative Dental Sciences Department, College of Dentistry, King Saud University, Riyadh 11545, Saudi Arabia; 2 Engineer Abdullah Bugshan Research Chair for Dental and Oral Rehabilitation, College of Dentistry, King Saud University, Riyadh 11545, Saudi Arabiawsaeed@ksu.edu.sa (W.S.S.); ibautista@ksu.edu.sa (L.S.B.); 3 Research Center, College of Dentistry, King Saud University, Riyadh 11451, Saudi Arabia; ialnofaiy@ksu.edu.sa (I.A.A.); mtandevera@ksu.edu.sa (M.A.T.D.V.)

**Keywords:** dimethacrylate, dental composite, TEGDMA, organic co-filler, microhardness

## Abstract

Dental resin composites (DRCs) have gained immense popularity as filling material in direct dental restorations. They are highly valued for their ability to closely resemble natural teeth and withstand harsh oral conditions. To increase the clinical performance of dental restorations, various fillers are incorporated into DRCs. Herein, the effect of incorporating pre-polymerized triethylene glycol dimethacrylate (P-TEGDMA) as a co-filler in varying proportions (0%, 2.5%, 5%, and 10% by weight) into bisphenol A-glycidyl methacrylate (BisGMA)/TEGDMA/SiO_2_ resin composite was investigated. The obtained DRCs were examined for morphology, rheological properties, degree of crosslinking (DC), Vickers microhardness (VMH), thermal stability, and flexural strength (FS). The results revealed that SiO_2_ and P-TEGDMA particles were uniformly dispersed. The introduction of P-TEGDMA particles (2.5 wt.%) into the resin composite had a remarkable effect, leading to a significant reduction (*p* ≤ 0.05) in complex viscosity, decreasing from 393.84 ± 21.65 Pa.s to 152.84 ± 23.94 Pa.s. As a result, the DC was significantly (*p* ≤ 0.05) improved from 61.76 ± 3.80% to 68.77 ± 2.31%. In addition, the composite mixture demonstrated a higher storage modulus (G′) than loss modulus (G″), indicative of its predominantly elastic nature. Moreover, the thermal stability of the DRCs was improved with the addition of P-TEGDMA particles by increasing the degradation temperature from 410 °C to 440 °C. However, the VMH was negatively affected. The study suggests that P-TEGDMA particles have the potential to be used as co-fillers alongside other inorganic fillers, offering a means to fine-tune the properties of DRCs and optimize their clinical performance.

## 1. Introduction

Dental resin composites (DRCs) are essentially composed of a binary framework featuring an organic matrix intertwined with an inorganic filler. The organic matrix is produced by the radical polymerization of dimethacrylates and polymerizes rapidly in the presence of oxygen and water. To improve the properties of dental restoration and mimic the phenomenal features of dental enamel, which contains more than 95 vol.% hydroxyapatite crystallites tightly packed into a complex microstructure, the organic matrix is filled with various inorganic fillers [1].

Bisphenol A-glycidyl methacrylate (BisGMA) is a commonly used dimethacrylate monomer in today’s commercial DRCs. When compared to its counterparts, BisGMA monomer exhibits lower shrinkage, higher modulus, and lower toxicity. However, the high viscosity of BisGMA is a great concern that inhibits the homogenous distribution of a high amount of filler. The addition of a high amount of filler into the monomer not only enhances the mechanical stability of DRCs but also reduces the shrinkage. Since the high viscosity of the BisGMA monomer inhibits the addition of a high amount of filler. Therefore, as a common practice, triethylene glycol dimethacrylate (TEGDMA) monomer is mixed due to its low viscosity as a diluent to balance the viscosity. However, the addition of TEGDMA increases the water sorption and curing shrinkage of the DRCs and negatively affects their durability [2]. Researchers are looking for alternative ways of reducing the viscosity of BisGMA or new dimethacrylates monomers with lower viscosities in order to reduce or eliminate the use of diluent. Previously, our group reported a structural analogous of BisGMA with low viscosity as an alternative [3]. Many other studies have also been reported on the evaluation of alternative monomers or structural analogs of BisGMA and address the issue of high viscosity [4,5].

The physio-mechanical characteristics of the DRCs are also impacted by the degree of crosslinking (DC). Generally, the higher DC results in high mechanical strength. However, factors like filler type, shape, size, and concentration also greatly influence the mechanical properties. The incorporation of nanoparticles (NPs) into DRCs has the potential to improve their characteristics including abrasion resistance, gloss retention, modulus, and flexural strength (FS) [6,7]. The effectiveness of these characteristics, however, is largely determined by how well the NPs are dispersed in the resin composite [8,9] because the uniform distribution of inorganic NPs in the resin is difficult due to their surface charge and high surface area. As a result, the NPs tend to aggregate, particularly at higher filler concentrations [10]. These aggregates contribute to material failure by acting as stress concentration points [11,12]. Therefore, in order to attain optimal dispersion of inorganic NPs and better interfacial adhesion, researchers have developed hybrid particles by encapsulating inorganic particles with polymer [13].

Surface microhardness is an important property of DRCs that is determined by the intrinsic strength and wear properties of a material. It measures the material’s ability to resist indentation and is closely related to the longevity of dental restorations. In addition, microhardness assessments have proven valuable in gauging the DC in polymer materials [14,15]. However, this correlation is not always proportional depending on the type and nature of the filler. Similarly, rheology (viscosity, visco-elasticity) and polymerization of DRCs are critical factors that have a significant role in clinical application. In particular, the viscosity of the commercial formulations is a highly important factor, as it has a direct impact on the ease of handling of DRCs, as well as influences the DC and polymerization kinetics during free radical reaction. This is due to lower mobility of chains and rate of termination as a result of higher viscosities [16,17]. The effects of resin on viscosity have been widely studied [18,19], research on how viscosity increases with the addition of filler particles, particularly at high filler concentration where interactions between particles become critical, is very limited.

Several new classes of DRCs based on the modification in filler particles (particle size, shape, nature) have been developed since the introduction of the first macrofill composite (10–50 µm) [20]. By varying the filler particle size and shape, the various features of the resulting DRCs could be altered, including surface roughness and polishing retention, mechanical strength, and toughness [21,22]. Owing to their high surface-to-volume ratio, small particles improve and increase the viscosity of resin by increasing filler-to-matrix interaction and reducing the filler loading [23,24]. Based on the size of reinforcing fillers, the RDC is characterized as nanofilled (5–100 nm), micro-filled (40–50 nm), hybrid (10–50 µm  +  40 nm), nanohybrid (0.6–1 µm  +  5–100 nm), microhybrid (0.6–1 µm  +  40 nm), and midifilled (1–10 µm  +  40 nm) [25]. Similarly, RDCs can be further classified as homogeneous or heterogeneous composites based on their preparation method [22]. Homogeneous composites involve the direct mixing of filler particles into the resin; however, the high surface area prevents the incorporation of high concentrations of filler. To cope with this issue, the heterogeneous composites are introduced with the filler particles coating with a pre-polymerized layer of resin, followed by incorporation into the non-polymerized resin formulation along with nano and micro inorganic fillers. The objective of using fillers coated with pre-polymerized resin is to enhance the inorganic filler content in the resulting DRC [26].

A number of nanohybrid DRC with pre-polymerized fillers are commercially available including Ceram.X^®^ (Dentsply DeTrey GmbH, Konstanz, Germany). However, in the current study, we have introduced the concept of pre-polymerized organic co-filler particles in combination with conventional inorganic filler (SiO_2_). Due to the hydrophilic nature of SiO_2_ and the presence of hydrogen bonding, NPs form irregular clusters by sticking together and significantly increasing the viscosity which negatively affects the filler loading. This study aimed to analyze the influence of integrating P–TEGDMA particles as co-filler on the viscosity, DC, thermal, and mechanical characteristics of BisGMA-based DRC. The null hypothesis was that the inclusion of P–TEGDMA would not influence the viscosity and DC. Furthermore, the thermal characteristics and microhardness of the resin composites will remain unchanged by the addition of TEGDMA.

## 2. Materials and Methods

### 2.1. Materials

BisGMA (98%), TEGDMA (>95%), azobisisobutyronitrile (AIBN; ≥98.0%), 2-(dimethylamino) ethyl-methacrylate (DMAEMA; 98%), 3-(tri-methoxy–silyl) propyl-methacrylate (γ-MPS; 98%), camphorquinone (CQ; 97%), tetraethyl orthosilicate (TEOS; 98%), and ethanol (EtOH, ≥99.8%) obtained from Sigma Aldrich (Taufkirchen, Germany). Ammonium hydro-oxide (NH_4_OH; 35%) was purchased from Fisher Scientific (Leicestershire, England). Materials in the study were used as received except AIBN which was purified by crystallization from ethanol before use.

### 2.2. Methods

#### 2.2.1. Preparation of TEGDMA Powder

**Figure 1:**
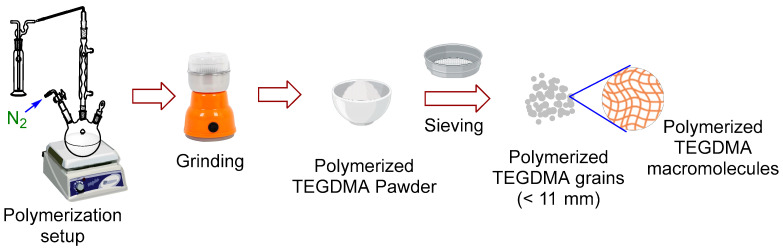
Schematic image of preparation of P–TEGDMA powder.

The TEGDMA powder particles were synthesized via a bulk polymerization reaction. The reaction was performed by thermal initiation using AIBN under inert N_2_ [24]. The synthesis process is depicted in Figure 1. In a reflex setup, the initiator (AIBN; 0.1 wt.% per monomer) was first dissolved in the monomer (TEGDMA) at room temperature and heated up to 70 °C, at which the polymerization reaction was left tell complete the conversion of the liquid phase to solid substance. The obtained solid material was subjected to cooling to obtain crosslinked polymer which was converted to powder with the help of an electrical crushing machine. The powder was then stained multiple times with ethanol and dried again for 24 h in a vacuum oven at 50 °C and kept in a tied container until use.

#### 2.2.2. Synthesis of Silanized Silica

The filler used was of silanized silica type and was obtained following a previously reported method [10]. Briefly, the TEOS silica precursor (45 mL) was dropwise added to a pre-cooled solvent at 5 °C in an ice bath that was considered the particle-size guidance solvent and consisted of water (40 mL), NH_4_OH (25 mL) and ethanol (250 mL). The reaction was brought to room temperature, and then additional TEOS (33 mL) in EtOH (250 mL) was supplied into the reaction mixture to continue for 8 h. For silanization, a 10 vol.% per TEOS of γ-MPS was incorporated and left overnight to complete the reaction. The salinized SiO_2_ (S-SiO_2_) was obtained by centrifuge process, followed by washing with EtOH and drying for 24 h.

#### 2.2.3. Experimental Dental Composites

The targeted model composites were designed as 1:1 (wt/wt) of organic matrix and filler. The organic matrix was fixed to 1:1 (wt/wt) BisGMA and TEGDMA monomers and included the initiation system that consists of 0.2 wt.% CQ and 0.8 wt.% DMAEMA. The filler portion was of S-SiO_2_ type, which was sequentially replaced with 0.0, 2.5, 5, and 10% of the synthesized pre-polymerized TEGDMA powder. The components were mixed following a recommended method [24] and the resulting composites were termed TRCx, where x is the P-TEGDMA (g) per 50 g filler, as summarized in Table 1. Briefly, the initiation system was introduced into the BisGMA and TEGDMA mixture, and then the corresponding fillers were suspended. The components were homogenized by mixing with a spatula, followed by mechanical stirring using a high-speed asymmetrical mixer at an rpm of 3000, for 2 min with 1 min pause and 3 repetitions. The mixture was stored below 8 °C until use.

The specimen to be analyzed for DC and VMH was prepared using a customized stainless-steel mold. After filling the mold was placed with a mylar strip and glass plate to prevent air effect during photocure. Samples were then cured for 60 s using a Bluephase unit (Ivoclar, Schaan, Lichtenstein) characterized with an intensity of 650 mW/cm^2^, wavelength range of 385–515 nm, and light output tip of 10 mm.

#### 2.2.4. Characterization

The filler dispersion, morphology, and elemental composition of the composites were evaluated by SEM (JSM-6610, JEOL, Tokyo, Japan) at 12 KV and 2000× and 5000× magnifications. The rheology test was carried out by using a rheometer (MRC-72, Anton Paar, Graz, Austria) under employing parallel plates oscillating mode (25 mm plate diameter and a 0.5 mm gap in between). The measurement was performed over the frequency range of 0.10–100 rad/s and at 25 °C.

The FTIR spectra were measured on a Nicolet-iS10 spectrometer (Thermo Fisher Scientific, Waltham, MA, USA) over a wavelength range of 4000–650 cm^−1^. The instrument is configured with an attenuated total reflection (ATR) measuring mode (diamond crystal). The DC was assessed in accordance with a standardized protocol [3]. Accordingly, the paste specimen was packed in a customized stainless steel mold (disc shape; 5 mm diameter × 2 mm thickness), which was sandwiched between two glass slides to provide a flat surface and prevent air void formation, then light cured for 60 s. The DC was obtained on the basis change of aliphatic C=C of methacrylate moiety with respect to the C=C bonds of aromatic rings assigned at 1638 and 1608 cm^−1^, respectively. Hence, the peak areas (A) were compared as shown in Equation (1) [3].

(1) $$DC\;\left(\%\right)=\left[1-\frac{{\left(\frac{A_{1638}}{A_{1608}}\right)}_{cured}}{{\left(\frac{A_{1638}}{A_{1608}}\right)}_{uncured}}\right]\times 100$$

Vickers microhardness device (Innovatest, FALCON– 500G2, Halesowen, UK) was used to analyze the Vicker’s hardness number (VHN) using 50 grams-force (gf) with 15 s of dwelling. The indentation was measured with a 40× lens of the microscope to calculate the diagonals. The VHN was calculated based on Equation (2). The obtained results of 3 replicates were statistically analyzed and reported as the mean and standard deviation (SD).

(2) $$VHN=\left(\frac{F}{D^{2}}\right)\times 1.854$$

where F (kilograms-force) and D (mm^2^) are the load and the indent area, respectively. Thermogravimetric analysis (TGA) was conducted on a TA instrument (Q50, New Castle, DE, USA) for samples (10–16 mg) heated from room temperature up to 800 °C at a rampe rate of 10 °C/min and under air environment.

The flexural strength (FS) of all the composite groups was measured by conducting a three-point bending (TPB) test as per ISO 4049 instruction [27], using Instron (Instron-5967, Norwood, MA, USA). The bar specimen (25 mm × 2 mm × 2 mm) was prepared by packing the resin mixture inside a silicon mold and a mylar strip and glass plate were placed on the mold to prevent air trap amid photo-polymerization. The specimens were then light cured at 3 points, keeping the same parameters as mentioned earlier with the Bluephase unit (Ivoclar, Schaan, Lichtenstein). After curing, the specimens were removed from the mold, trimmed, and polished with 600-grit SiC paper. The dimensions were recorded with digital caliper (Digimatic, Mitutoyo Corp., Kawasaki, Japan). The bar samples were kept in distilled water (24 h at 37 °C) prior to TPB analysis. The TPB test was performed at a crosshead speed of speed of 0.5 mm/min until fracture. The maximum fracture force (F) was obtained for all groups, and the FS (σ_f,_ MPa) was calculated as per Equation (3).

(3) $$\sigma_{f}=\frac{3Fl}{2bh^{2}}$$

where σ_f_ = FS, F = maximum fracture force, l = gap between support points (20 mm), b = width of sample and h = height of sample. 

The obtained data were statistically evaluated by one-way ANOVA and Tukey’s post hoc analysis using SPSS (V 21.0, Chicago, IL, USA) by considering a *p* value of 0.05 as significant.

## 3. Results and Discussion

**Figure 2:**
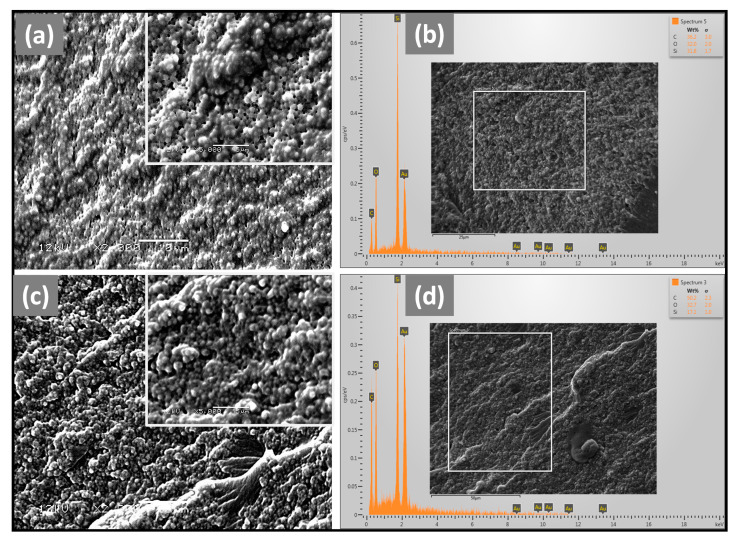
SEM micrographs and EDX analysis DRCs with and without P-TEGDMA: (**a**) TRC0 at low and high magnification (inset), (**b**) EDX analysis of TRC0, (**c**) TRC10 at low and high magnification (inset), (**d**) EDX analysis of TRC10.

The morphological of the composites with and without P-TEGDMA and the dispersion of SiO_2_ and P-TEGDMA in the composites were evaluated by scanning electron microscopy (SEM). In addition, the elemental analysis was also conducted with energy dispersive X-ray (EDX) as shown in Figure 2a–d. The SEM images showed a uniform distribution of SiO_2_ and P-TEGDMA. From Figure 2a, it can be seen that the SiO_2_ particles are coated with resin and uniformly distributed as they have a spherical shape and uniform particle size; however, microvoids could be observed in the structure, which may result in poor interfacial interaction among the filler and the matrix. Under stress, these microvoids may promote the formation of cracks and spread freely as a brittle material. In contrast, the addition of P-TEGDMA (Figure 2c) resulted in a more compact morphology and improved the interaction between filler and resin. The densely packed P-TEGDMA particles embedded in the composite may limit the mobility of the polymeric chain segments and may absorb the deformation energy under stress applications. The elemental composition of the composites was confirmed by EDX as depicted in Figure 2b,c. The amount of Si in the TRC10 (Figure 2d) is reduced while the C content is increased by adding P-TEGDMA.

**Figure 3:**
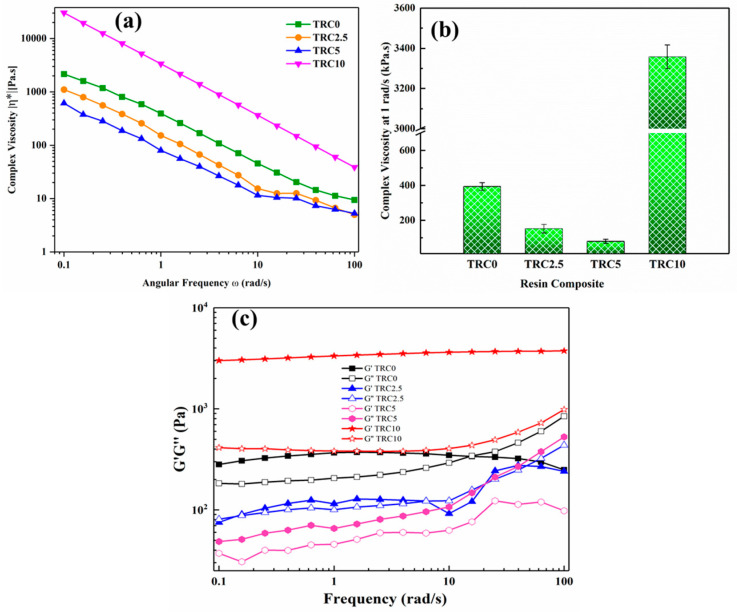
Viscoelastic properties of BisGMA/TEGDMA/SiO_2_ dental composites: (**a**) complex viscosity (0.1 to 100 rad/s), (**b**) complex viscosity at 1 rad/s, (**c**) elastic and storage modulus (G′ and G″).

The viscoelastic response of all the test group mixtures (TRC0, TRC2.5, TRC5, and TRC10) are depicted in Figure 3a–c. Figure 3a shows the complex viscosities of all the groups between the angular frequency of 0.1 and 100 rad/s. It could be observed that all the groups demonstrated strong non-Newtonian behavior: the complex viscosity decreased with increasing frequency [28]. The decrease in viscosity could be attributed to thinning behavior caused by the disruption of intermolecular interactions between resin matrix constituents induced by hydrogen bonding and interactions as a result of a high shear rate [29]. To study the influence of TEGDMA particles on the viscosity of the DRC mixtures, the complex viscosity at 1 rad/s is selected to compare various groups as shown in Figure 3b. The pristine composite mixture with no TEGDMA particles (TRC0) resulted in the highest viscosity (393.84 ± 21.65 Pa.s). The additional 2.5 wt.% P–TEGDMA particles (TRC2.5) resulted a significant reduction (*p* ≤ 0.05) in the viscosity (152.84 ± 23.94 Pa.s) followed by TRC5 (79.956 ± 11.75 Pa.s). However, the high concentration of TEGDMA particles (TRC10) has a negative impact on the viscosity, which resulted in a significantly higher (*p* ≤ 0.05) viscosity (3358 ± 58.56 Pa.s). This behavior of the resin mixture demonstrates that the incorporation of prepolymerized TEGDMA influenced the flow properties of the composite mixture, thus rejecting one of the null hypotheses. The reduced viscosity may be associated with the plasticizing behavior of P–TEGDMA enhancing polymer chain mobility. On the contrary, adding a higher percentage of P–TEGDMA (10 wt.%) transformed the mixture into a gel as a result of the saturation of monomer chains, inhibiting further absorption of P–TEGDMA filler and hindering the mobility of polymeric chains [30]. The order of the viscosity for the test groups was recorded as TRC10 > TRC0 > TRC2.5 > TRC5. The least viscosity was observed for TRC5 (79.956 ± 11.75 rad/s).

The viscoelastic response of the test groups and the corresponding curves of storage (elastic-response) modulus (G′) and loss (viscous-response) modulus (G″) against angular frequency are illustrated in Figure 3c. It can be inferred that the G′ was greater than G″ at the early age in TRC0 at a certain frequency range (approx.0.1 to 10 rad/s) showing an elastically dominant (elastic solid) behavior. However, at higher frequencies, the G′ crossover G″ where G′ = G″. The crossover-point measures the equilibrium between G′ and G″ and is considered the transitional stage of solid and liquid [31]. After the crossover frequency, the G″ > G′, showing a dominating viscous and liquid-like behavior. This behavior may be associated with the rapid thixotropy, the entrapment of the SiO_2_ nanoparticles within the matrix. The complex viscosity reduces with increasing angular frequency due to the adjustment of chain alignment and relaxation [32,33]. At high frequencies, the resin mixture demonstrated a reverse trend (G″ > G′) showing a predominantly viscous behavior. The addition of a low concentration of P–TEGDMA particles (TRC2.5) along reduction in viscosity, reduced the crossover frequency as well. In other words, the crossover frequency is a measure of the relaxation time (reciprocal of angular frequency values at the crossover point where the G′ and G″ intersect) which quantifies a polymer’s viscoelastic character. The relaxation time (t) corresponds to the elastic behavior of polymeric materials. High relaxation time represents the elastic response of the polymeric materials [34]. In the case of TRC2.5, the lower crossover frequency demonstrates a longer relaxation time which shows an increase in elastic characteristic of the composite mixture. All the other groups (TRC5 and TRC10) demonstrated higher G′ value than G″ not reaching a crossover, representing a gel-like nature of the mixtures [31]. However, at a high concentration of P–TEGDMA (TRC10), the elastic component (G′) was significantly dominant showing a highly solid-like nature of the mixture. Notably, as the P–TEGDMA content is increased, a noticeable shift between G′ and G″ becomes evident, highlighting the considerable impact of filler concentration on the storage characteristics of the blend. The viscoelastic response of the TRC 5 and TRC 10 resin mixtures reveals a consistent pattern: G′ surpasses G″ across all frequencies. This signifies the blend’s stability even at higher frequencies, affirming the prevalence of elastic nature of the mixture over its viscous characteristics.

**Figure 4:**
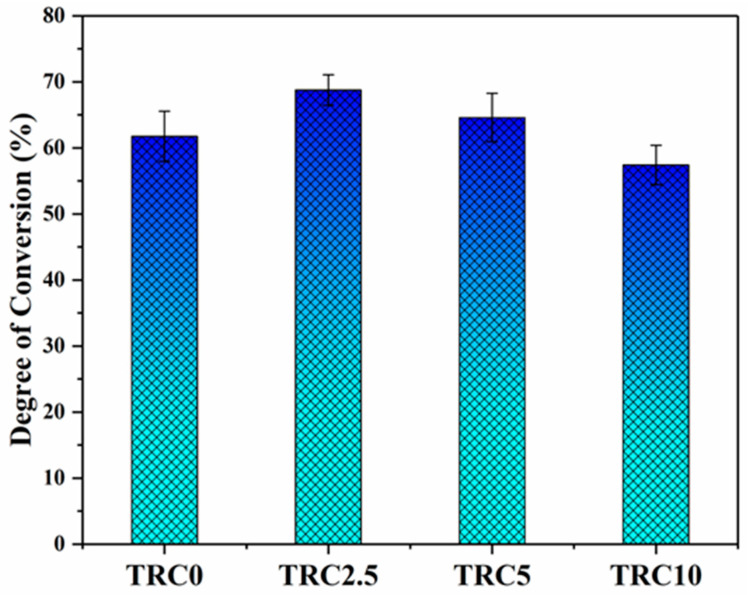
Effect of TEGDMA particles on the degree of conversion.

Figure 4 depicts the effect of P–TEGDMA co-filler on the DC of the various groups. The pristine composite group (TRC0) demonstrated a DC value of 61.76 ± 3.80%. The addition of 2.5 wt.% of P–TEGDMA particles considerably (*p* ≤ 0.05) increased the DC (68.77 ± 2.31%) rejecting the null hypothesis. Further addition of P–TEGDMA (TRC5) slightly reduced the DC (64.6 ± 3.67%), however, the reduction in DC was statistically not significant (*p* ≥ 0.05). The addition of a high concentration of TEGDMA particles (TRC10), demonstrated a significantly low (*p* ≤ 0.05) DC (57.43 ± 2.30%). The reduction in DC of the composite groups may be correlated with the reduction in viscosity at lower concentrations of P–TEGDMA particles (TRC2.5, TRC5). However, the higher concentration of P–TEGDMA particles in the composite (TRC10), negatively affected the DC of the composite causing a significant reduction (*p* ≤ 0.05). It is evident from previous studies that, materials with a lower viscosity result in higher DC than those with a higher viscosity and the current results are in line with the literature [35]. The decrease in DC due to high viscosity can be ascribed to the restriction of monomer chain mobility and a reduction in chain propagation during the polymerization process [36]. The reduced viscosity supports enhanced chain mobility and the widespread dispersion of free radicals, thus bolstering the radical chain polymerization process and yielding a higher DC. Consequently, DRCs characterized by low viscosity facilitate the diffusion of reactive groups, facilitating the crosslinking reaction and leading to high DC [37]. However other factors including the constituent of the dental composite, the nature of each constituent, and their interaction with each other also play a significant role in DC [38,39].

**Figure 5:**
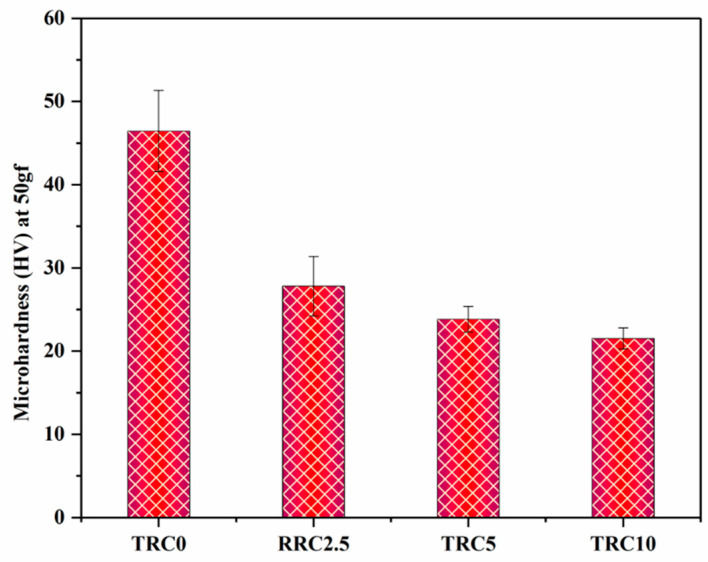
Microhardness of the composite groups with and without TEGDMA particles.

Microhardness is a measure of resistance to penetration or permanent deflection on a surface and is important in predicting the wear properties and durability of clinical restorations [40]. Vickers microhardness (VMH) is generally used for evaluating the surface hardness of dental composites [41,42]. Figure 5 illustrates the VMH of all the composite groups with and without P–TEGDMA particles. The results revealed that the VMH of the composite groups gradually decreased with increasing P–TEGDMA particle concentration. The composite group with no P–TEGDMA (TRC0) exhibited the highest VMH (46.44 ± 4.89 HV). However, the incorporation of a small percentage of P–TEGDMA (TRC2.5) as a co-filler significantly (*p* ≤ 0.05) reduced the VMH (27.81 ± 3.58 HV). A further increase in the concentration of P–TEGDMA particles (TRC5, TRC10) followed the same pattern of reduction in VMH (23.83 ± 1.55 HV, 21.52 ± 1.27 HV, respectively); however, the reduction in the VMH at high concentration of P–TEGDMA was not significant (*p* > 0.05). TRC10 resulted in the lowest VMH value (21.52 ± 1.27 HV) among the P–TEGDMA composite groups.

Generally, in DRCs the DC is one of the main factor directly influencing the hardness, because the high DC induce a densely compacted network due to crosslinking [43,44]. Nonetheless, the key factors influencing surface hardness are the type, particle size, and concentration of the reinforcing filler. Previous studies have also shown that organic pre-polymerized fillers tend to exhibit lower hardness compared to inorganic fillers [40]. Hence, it can be deduced that the decrease in the VMH values of the experimental DRCs in this study might be linked to the organic characteristics of the TEGDMA co-filler [26]. Because, despite the increase in the DC, the VMH is negatively affected. This could be regarded as a clinical constraint for these composites because several other properties, including durability, tribological characteristics, surface roughness, and plaque accumulation, are also impacted by VMH [40]. Therefore, a trade-off between various properties can be achieved by investigating various combinations of organic and inorganic fillers, given that certain DRCs may showcase robust surface hardness while exhibiting compromised flexural characteristics [45]. Therefore, VMH may not be considered the sole indicator of predicting the durability of the clinical performance of DRCs. Moreover, opalescence is an optical phenomenon tied to the aesthetical aspects of DRCs. It is induced by the light scattering as a result of the deviation in the refractive indices of both the resin matrix and fillers. This effect leads to the appearance of a blue artifact in light reflection while orange/brown under light transmittance [46]. The opalescence can be achieved by fine-tuning the refractive index and introducing inorganic fillers like ZrO_2_, Al_2_O_3_, and TiO_2_ [47]. The desired level of opalescence can be accomplished by incorporating organic groups into the silica filler to obtain a hybrid organic–inorganic filler, enhancing the filler/monomer ratio, or decreasing the particle size (down to 100 nm in diameter) [47].

**Figure 6:**
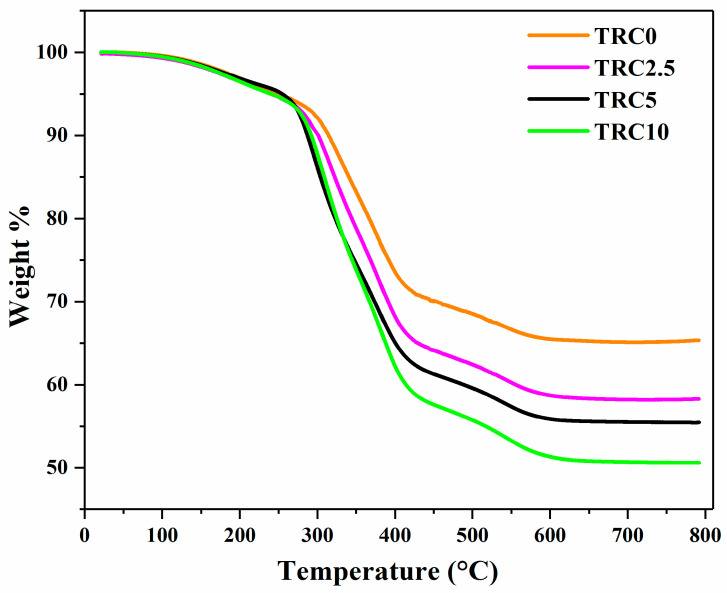
Thermal analysis of the pristine and various P-TEGDMA composite groups.

Figure 6 shows the characteristic TGA thermograms of the model composites, with and without P–TEGDMA particles. A two-stage degradation process was observed for all composite groups. In the first stage, a small weight was lost in the temperature range of 155 to 260 °C, probably due to the evaporation of absorbed gases, volatile molecules, moisture, and breakdown (homolytic) of certain chemical bondings within the polymeric structure [48]. The introduction of a higher content of P–TEGDMA led to an increase in the initial decomposition temperature as it shifted from 305 °C for TRC0 to 410 °C for TRC10. The significant weight loss during this stage can be linked to the disintegration and backbone chain decomposition of the polymer [49]. The second stage degradation was observed between 400–590 °C, which may be due to the dissociation of benzene aromatic rings within the crosslinked BisGMA, known for their high C-C bond energy [50,51]. The obtained residual char reduced significantly as the P–TEGDMA content increased, with TRC0 exhibiting the highest residual char and TRC10 resulting in the lowest residual char. This reduction in residual char might be correlated with the lower concentration of SiO_2_ NPs in TRC10. Moreover, the enhanced decomposition temperature from 410 °C to 430 °C can be attributed to the high crosslink density and the prevalence of the Benzene pendant groups.

Table 2 summarizes the mean and standard deviation values FS (MPa) for the various composite groups with and without P-TEGDMA. From the results, it can be observed that the FS of the composite was reduced initially by the addition of P–TEGDMA (2.5 wt.%) in TRC2.5. However, increasing the concentration of P–TEGDMA, enhanced the FS with TRC10 demonstrating the highest FS (94.72 MPa). The increase in the FS of TRC10 may be associated with better filler-to-resin interaction and compact microstructure due to packing P-TEGDMA to fill the microvoids. These results may be corroborated with SEM micrographs and complex viscosity analysis as given in Figure 2 and Figure 3, respectively. The compact packing of P-TEGDMA particles embedded in the composite may limit the mobility of the polymeric chain segments and may absorb the deformation energy under stress applications.

## 4. Conclusions

The effect of various concentrations of P–TEGDMA particles as co-filler in combination with SiO_2_ on viscosity, DC, micro-hardness, and thermal stability has been investigated. The addition of P–TEGDMA particles significantly reduced the complex viscosity of the resin composite system and the DC was increased. Moreover, P–TEGDMA enhances filler-to-resin interaction by resulting in a more compact composite as compared with pristine, thus enhancing the flexural strength at high P–TEGDMA loading. Similarly, the thermal stability of the DRCs were enhanced with increasing P-TEGDMA contents. However, the microhardness was reduced with the addition of P–TEGDMA particles which is a limitation of the current study. The microhardness and other mechanical properties could be balanced by combining P–TEGDMA with other inorganic fillers to achieve the desired properties for specific conditions. From this study, it can be concluded that P–TEGDMA particles have the potential to be used as a co-filler alongside inorganic counterparts to balance the properties of resin systems. However, further research and adaptation are required for the effective use of P–TEGDMA particles in dental composites.

## Figures and Tables

**Table 1:** Composition of the prepared model composites.

Composite	Filler (wt.%)		Resin Matrix (wt.%)		Initiation System (wt.%)	
	S-SiO_2_	P-TEGDMA	BisGMA	TEGDMA	Initiator (CQ)	Accelerator (DMAEMA)
TRC0	50	0	24.5	24.5	0.2	0.8
TRC2.5	47.5	2.5	24.5	24.5	0.2	0.8
TRC5	45	5	24.5	24.5	0.2	0.8
TRC10	40	10	24.5	24.5	0.2	0.8

**Table 2:** FS of various composite groups.

S No.	Group Name	FS(MPa) + (SD)
1	TRC0	89.31 (0.89)
2	TRC2.5	72.48 (3.86) ^a^
3	TRC5	77.21 (2.17) ^ab^
4	TRC10	94.72 (3.06) ^abc^

Note: Superscripts a, b, and c represent significant differences from TRC0, TRC2.5, and TRC5, respectively.

## Data Availability

No new data were created or analyzed in this study. Data sharing is not applicable to this article.
